# Evaluation of fecal mRNA reproducibility via a marginal transformed mixture modeling approach

**DOI:** 10.1186/1471-2105-11-13

**Published:** 2010-01-07

**Authors:** Nysia I George, Joanne R Lupton, Nancy D Turner, Robert S Chapkin, Laurie A Davidson, Naisyin Wang

**Affiliations:** 1 National Center for Toxicological Research, U.S. Food and Drug Administration, Jefferson, AR 72079, USA; 2 Program in Integrative Nutrition & Complex Diseases, Texas A&M University, College Station, Texas 77843-2253, USA; 3 Department of Statistics, University of Michigan, Ann Arbor, MI 48109-1107, USA

## Abstract

**Background:**

Developing and evaluating new technology that enables researchers to recover gene-expression levels of colonic cells from fecal samples could be key to a non-invasive screening tool for early detection of colon cancer. The current study, to the best of our knowledge, is the first to investigate and report the reproducibility of fecal microarray data. Using the intraclass correlation coefficient (ICC) as a measure of reproducibility and the preliminary analysis of fecal and mucosal data, we assessed the reliability of mixture density estimation and the reproducibility of fecal microarray data. Using Monte Carlo-based methods, we explored whether ICC values should be modeled as a beta-mixture or transformed first and fitted with a normal-mixture. We used outcomes from bootstrapped goodness-of-fit tests to determine which approach is less sensitive toward potential violation of distributional assumptions.

**Results:**

The graphical examination of both the distributions of ICC and probit-transformed ICC (PT-ICC) clearly shows that there are two components in the distributions. For ICC measurements, which are between 0 and 1, the practice in literature has been to assume that the data points are from a beta-mixture distribution. Nevertheless, in our study we show that the use of a normal-mixture modeling approach on PT-ICC could provide superior performance.

**Conclusions:**

When modeling ICC values of gene expression levels, using mixture of normals in the probit-transformed (PT) scale is less sensitive toward model mis-specification than using mixture of betas. We show that a biased conclusion could be made if we follow the traditional approach and model the two sets of ICC values using the mixture of betas directly. The problematic estimation arises from the sensitivity of beta-mixtures toward model mis-specification, particularly when there are observations in the neighborhood of the the boundary points, 0 or 1. Since beta-mixture modeling is commonly used in approximating the distribution of measurements between 0 and 1, our findings have important implications beyond the findings of the current study. By using the normal-mixture approach on PT-ICC, we observed the quality of reproducible genes in fecal array data to be comparable to those in mucosal arrays.

## Background

Microarray techniques have changed the practice of detecting messenger RNA (mRNA) expression of a single gene to the current stage of simultaneously measuring the expression of thousands of genes. Daily improvement in this technology also stimulates techniques that lead to new bioassays. Among them, and of particular interest, is a recent development that enables the collection of genomic information from exfoliated colonocytes in fecal matter. It is known that early detection of cancerous colon cells results in high cure and survival rates among colon cancer patients. However, people tend to shy away from invasive procedures such as the colonoscopy. Consequently, it is of great interest to develop non-invasive early detection instruments. Although evidence exists in the fecal platform that partially degraded mRNA in fecal samples can produce meaningful measurements[1], and Davidson *et al*. [2] and Kanaoka *et al*. [3] suggest that it is possible to isolate intact fecal eukaryotic mRNA, it is unknown whether one can expect the same quality from the large amount of fecal microarray data. The current study, to the best of our knowledge, is the first one that investigates and reports the reproducibility of fecal microarray data. In a proof-of-principle study conducted by human nutrition scientists at Texas A&M University, one main task is to find out whether one can expect the same level of reproducibility in the fecal platform as that observed in the mucosal platform where biological samples were taken from colon cells. Because of biological variation, two gene expression values of the same gene taken from the same subject are most likely not the same. In order to determine if one can successfully obtain the same findings when an experiment is repeated, it is important to investigate whether the gene expression levels of a gene from the same subject behave more similarly to each other than to those of the same gene from different subjects. The signal is strongest and the reproducibility is highest when the outcomes can be perfectly repeated when a different set of measurements are taken from the same subjects. It is expected that due to mRNA degradation, a larger proportion of genes in the fecal platform would possess no or lower reproducibility than those in the mucosal platform. However, it is of interest to understand the quality of those genes which are not degraded in the fecal platform.

Generally, replicates are samples collected from the same subject that are processed separately and independently after sample collection. Our replicates differ because the "same" biological samples are separately processed only right before the hybridization. The former "replicates" are often collected to evaluate the quality of microarray techniques, while we are truly interested in biological reproducibility at the subject level. This subtle difference is particularly important; some genes could be preserved in one sample but are degraded in another even when both samples are from the same subject. It is the genes with low possibility to be degraded that we are interested in. While we focus only on subject to subject variation, we acknowledge that there are other types of replication in gene expression data[4].

In order to assess the agreement between measurements from microarray data collected from the same subject, we use the intraclass correlation coefficient (ICC) as a reliability index. The use of ICC in genomic study was promoted by Carrasco and Jover[5].

Under each platform, we compute a single ICC value for each gene. One key advantage of ICC as a statistical tool for evaluating reproducibility for different platforms/instruments is that it does not require two platforms/instruments to be evaluated under the same treatment design. In most biological experiments, researchers tend to conduct the second experiment with modifications and improvements rather than simply to repeat what has been done before. Consequently, a statistical tool for evaluating reproducibility has to have the flexibility to accommodate this common practice. In order to fulfill this requirement, the ICC values were computed after removing the treatment effects. The single index recorded per gene uses variance components analysis to compare the measurement-similarity for samples taken from the same subject/rat versus the measurement-similarity for samples taken from different subjects/rats. We report the methodology for calculating ICC in the Methods subsection.

The larger the value of ICC, the more differentiation among measurements collected from different biological samples relative to that among readings collected from the same biological material. An ICC value near 1 signifies a strong indication of reproducibility and agreement between experiments. If the ICC is near 0, then within-subject variance is relatively large compared to between-subject variance and it is likely that one cannot obtain the same expression level in a repeated experiment.

In both the mucosal and the fecal genes, we observe at least a small proportion of genes that always have low reproducibility; their existence results in a mixture model for the distribution of ICC values. It is common practice to use finite mixture modeling in bioinformatics research. The reasons tend to be twofold: to accommodate measurement heterogeneity and to identify potentially meaningful subgroups. The most popular approach is the use of finite normal mixtures [6-9]. Allison *et al*. and Ji *et al*. use beta-mixture modeling to describe distributional properties of different genes' correlation coefficients[10,11]. Like measurements of ICC, the values of correlation coefficients are between 0 and 1. For the same type of data, McLachlan *et al*. prefer the use of normal-mixture distributions which eliminates the (0,1)-range constraint[8].

In a study comparing the fecal and mucosal bioassay platforms, we obtained different proportions for the mixture components when we modeled the probit transformed ICC (PT-ICC) values with a two-component normal-mixture distribution and when we modeled the ICC values with a two-component beta-mixture distribution. It was our conjecture that, considering the boundary problem of beta distribution modeling, the normal-mixture modeling might be less sensitive toward model mis-specification. We observed the lower component of the beta mixture to be strictly decreasing with the density *f*(*y*|*α,β*) approaching infinity as *y *approaches 0. This phenomenon likely caused the maximum likelihood estimate (MLE) of *β*-parameters to be unstable. We conduct a sequence of numerical studies to compare the two approaches.

Our ultimate goal is to select the better of the two systems to ascertain whether the "reproducible" component in the fecal array samples share similar properties to those of the mucosal array samples.

## Results

### Data sets

Gene expression levels from the colon mucosal and fecal data samples were collected using CodeLink microarrays (30 oligonucleotide target probe, single color labeling system). The main dataset under study here consisted of 2171 genes for the fecal data and 2241 genes for the mucosal data. Due to the fact that the bioassays that were used to extract fecal mRNA were developed later, the mucosal data we used were collected much earlier in a different experiment. In fact, we did not have access to the original muscosal dataset. We were able to use the available summary statistics to produce ICC measurements. All measurements (fecal and mucosal) were collected from Spraque Dawley rats.

#### Fecal Data

The fecal array data were collected from rat fecal samples in a study designed to explore the effect that diet has on genes being differentially expressed after exposure to carcinogen/radiation. A normalization procedure was developed[12]. Rats in the study were exposed to carcinogen azoxymethane (AOM) and randomly assigned to one of four different treatments resulting from a 2 × 2 factorial design. The two experimental factors were diet - fish oil/pectin (D1) and corn oil/cellulose (D2), and radiation - with radiation exposure (IRT) and without radiation exposure (RCT). Fecal samples were collected 14 weeks after the last exposure to carcinogen AOM. There were 7, 6, 8, and 7 bioarrays collected under IRT-D1, IRT-D2, RCT-D1, and RCT-D2, respectively. Genes that were not disqualified with at least 3 usable replicates were kept.

#### Mucosal Data

Rats used in the study to obtain mucosal array data were randomly assigned in a 3 × 2 × 2 factorial experiment to a treatment with diet, exposure, and time points as factors[13]. Corn oil/*n*-6 polyunsaturated fatty acid (PUFA) or fish oil/*n*-3 PUFA or olive oil/*n*-9 monounsaturated fatty acid (MUFA) was used as the dietary fat source; carcinogen AOM or saline was used as the exposure source; time points were either 12 hours or 10 weeks after the first injection. The units were terminated at the appropriate time point in order to remove the mucosal layer from each colon so that RNA could be extracted from the mucosal samples. The numbers of arrays for corn, fish, and olive oil diets under AOM or saline treatments were (7, 7, 6) and (7, 6, 7), respectively for the 12-hour study and were (12, 10, 8) and (7, 9, 7), respectively for the 10-week study.

#### Matched Subset

To address the issue of reproducibility for a finite list of common genes between the platforms, we conducted an additional study referred to as the "matched subset" throughout. We were able to retrieve the NCBI gene information from the mucosal experiments and used them to create a matched subset in which the two subsets (fecal and mucosal) were collected from the same genes. Each subset contains 1029 measurements.

### Preliminary Application to the Main Dataset

The original ICC values were fitted with a two-component beta-mixture using an EM algorithm, producing the following density estimation for the fecal and mucosal data,  and , respectively:

and

We obtained the following estimated two-component normal-mixture densities,  and , for the probit transformed fecal and mucosal ICC measurements, respectively:

and

**Figure 1:**
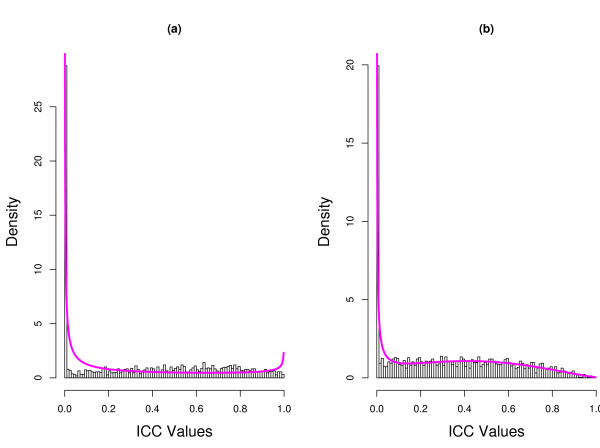
**Histogram of ICC values**. The density of the fitted two-component beta-mixture to the (a) fecal data and (b) mucosal data is superimposed.

**Figure 2:**
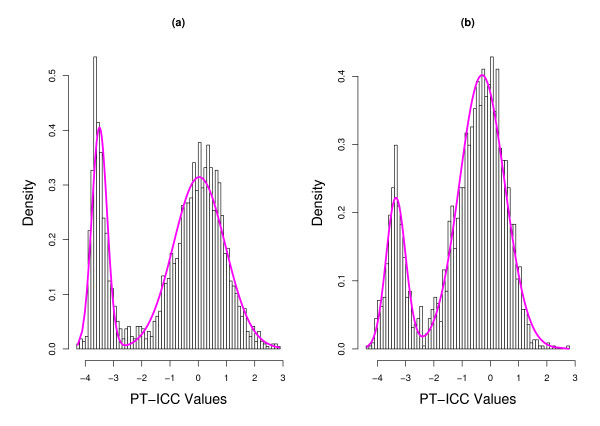
**Histogram of PT-ICC values**. The density of the fitted two-component normal-mixture to the (a) fecal data and (b) mucosal data is superimposed.

**Figure 3:**
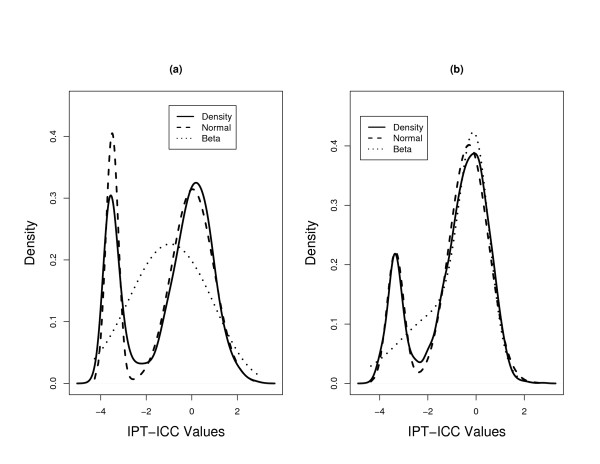
**Density estimates of the probit transformed ICC values for (a) fecal data and (b) mucosal data**. The solid, dashed, and dotted lines correspond to the kernel-based, the normal-mixture based, and the beta-mixture based density estimates.

The observation of the difference in proportion estimates for fecal and mucosal data leads us to question the accuracy of the two fits. It is unclear what the proportion of reproducible genes (upper component of the two mixtures) for the fecal samples should be, 0.50 or 0.72? Unfortunately, the answer to this question depends on the mixture model we use to fit the data. It is well known that when *α *< 1 (*β *< 1), values of the beta distribution strictly increase to infinity at the lower (upper) endpoint. We find *α *is much smaller than 1 with the lower components of the beta mixtures for both datasets. This phenomenon is easily seen in the graphs displayed in Figure 1 where we plot the fitted beta-mixture superimposed on the histogram of ICC values for the fecal and mucosal data. Because the beta distribution has such a boundary issue, we suspect that a simple violation of the distributional assumption near the boundary could have profound effects on maximum likelihood estimates. In comparison, the fitted normal-mixture superimposed on the histogram of PT-ICC values is plotted in Figure 2. It is worth noting that the visual evaluation of Figures 1 and 2 might not be helpful to the comparisons of these two modeling approaches. We investigate the veracity of the comparisons with numerical studies. In light of the numerical outcomes from our Monte Carlo investigation, we plotted three estimated density functions in Figure 3. The solid curves in each plot of Figure 3 provide the kernel estimated density functions of the fecal and mucosal PT-ICC values. The estimated density functions based on the normal-mixture models are given by the dashed lines. Finally, the estimated density function calculated using the transformation theory gave the estimated density functions of PT-ICC values shown by the dotted lines. Even though not perfectly, the kernel density estimates and the normal-mixture based estimates correspond roughly well with each other. However, the transformed beta-mixture based density estimates misfit the lower mixture component for the mucosal data. For fecal data, this approach almost concluded that there was a single component - a feature which could not be clearly seen in Figure 1.

### Monte Carlo Assessments

To investigate the sensitivity of each of the two mixture modeling approaches to distributional mis-specification, we conduct Monte Carlo simulation studies to mimic what we observed in the fecal and mucosal microarray data sets. Simulation for the fecal data is described as follows:

Simulation scenario #1: Data Generated from Beta-mixtures, Fit with Normal-mixtures

*(1) *Generate *Y*_1_, ..., *Y*_*n *_from  = 0.7 *Beta*(2.6, 1.7) + 0.3 *Beta*(0.2, 0.8).

*(2) *Transform *Y*_1_, ..., *Y*_*n *_using the probit transformation and fit the PT-ICC measurements with a two-component normal-mixture model.

Simulation scenario #2: Data Generated from Normal-mixtures, Fit with Beta-mixtures

*(1) *Generate *X*_1_, ..., *X*_*n *_from  = 0.7 *N *(0.04, 0.8) + 0.3 *N *(-3.5, 0.07).

*(2) *Transform *X*_1_, ..., *X*_*n *_using the inverse probit transformation and fit the ICC data with a two-component beta-mixture model.

We repeated each simulation *s *= 250 times for sample size *n *= 1600 and used the EM algorithm to obtain the estimates of corresponding parameters. The steps above were repeated for the mucosal dataset where the beta random variables were generated from  = 0.8 *Beta*(2.3, 2.3) + 0.2 *Beta*(0.3, 1.3) and the normal random variables were generated from  = 0.8 *N *(-0.3, 0.6) + 0.2 *N *(-3.3, 0.1).

We could not compare the outcomes of simulations #1 and #2 directly when the estimated parameters were for normal-mixtures and beta-mixtures, respectively. To ease the comparisons, we chose to transform the resulting estimates in simulation #2 so that the outcomes correspond to means and variances of distributions that would give observations on the whole real line, and then produced the Monte Carlo statistics corresponding to the two components. Summary statistics for simulation scenarios #1 and #2 are presented in Tables 1 and 2, respectively. We identified the targeted parameter values in each scenario as "Truth" and reported the Monte Carlo mean, bias, standard deviation, and square-root of mean squared error (RMSE) of the estimates. When comparing the true parameters with the estimates obtained from the fit of the assumed distribution, we find that summary statistics from fitting transformed normal random variables with a beta-mixture closely resemble the phenomenon observed when analyzing the fecal and mucosal data. Namely, it is the case that although the true proportions for the upper components of the fecal and mucosal data are 0.7 and 0.8, respectively, estimates of *π*_*U *_resulting from the fit of two-component beta distribution average 0.5. In contrast, modeling the simulated PT-ICC by normal-mixtures when the ICC values were generated from the beta-mixtures, as described in simulation scenario #1, is much less sensitive toward the distributional mis-specification. This led us to believe that the use of the two-component normal-mixture model on PT-ICC is the more reliable approach of the two. We further analyzed the simulated outcomes and compared the sensitivity of each modeling approach toward distributional mis-specification through performing goodness-of-fit tests against assumed models.

**Table 1:** Summary Statistics of Simulation Scenario #1

*Data Generated from Beta-mixtures, Fit with Normal-mixtures*						
**Dataset**						

**Fecal**	Truth	0.700	0.328	0.446	-1.771	3.330
	Mean	0.725	0.302	0.440	-1.951	3.321
	Bias	0.025	-0.026	-0.006	-0.180	-0.009
	Std Dev	0.018	0.023	0.028	0.152	0.283
	MSE	0.031	0.035	0.029	0.235	0.283
**Mucosal**	Truth	0.800	-0.033	0.391	-2.090	2.722
	Mean	0.816	-0.049	0.398	-2.254	2.823
	Bias	0.016	-0.016	0.007	-0.164	0.101
	Std Dev	0.015	0.022	0.022	0.157	0.272
	RMSE	0.022	0.027	0.023	0.227	0.290

Summary statistics of simulation scenario #1. Monte Carlo mean, bias, standard deviation, and square-root MSE (RMSE) of upper mixture proportion *μ*_*U*_, upper mixture mean *μ*_*U *_and variance , and lower mixture mean *μ*_*L *_and variance  from scenario #1.

**Table 2:** Summary Statistics of Simulation Scenario #2

*Data Generated from Normal-mixtures, Fit with Beta-mixtures*						
**Dataset**						

**Fecal**	Truth	0.700	0.328	0.446	-1.771	3.330
	Mean	0.453	0.282	0.521	-1.995	3.409
	Bias	-0.247	-0.046	0.075	-0.224	0.079
	Std Dev	0.010	0.036	0.032	0.050	0.138
	RMSE	0.247	0.059	0.082	0.229	0.159
**Mucosal**	Truth	0.800	-0.033	0.391	-2.090	2.722
	Mean	0.527	-0.149	0.387	-1.691	2.546
	Bias	-0.273	-0.116	-0.004	0.399	-0.176
	Std Dev	0.011	0.031	0.023	0.049	0.111
	RMSE	0.273	0.120	0.023	0.402	0.208

Summary statistics of simulation scenario #2. Monte Carlo mean, bias, standard deviation, and square-root MSE (RMSE) of upper mixture proportion *μ*_*U*_, upper mixture mean *μ*_*U *_and variance , and lower mixture mean *μ*_*L *_and variance  from scenario #2.

Precisely, for each simulated data set, we let the null hypothesis, *H*_0_, be that the observed ICC (or PT-ICC) values were from the assumed model. We then compared the observed and expected counts of observations within *K *bins, where *K *= 5, 8, and 12, using Pearson's chi-square goodness-of-fit test with significance level *α *= 0.05 and *k *- 1 degrees of freedom. The exact procedure of the test is described in the Methods subsection. Analysis of goodness-of-fit test statistics resulting from the simulation studies are given in Table 3. Ideally, if the *H*_0 _was true, there should be no more than 5% chance to reject the *H*_0 _when *α *= 0.05. Except when *K *= 5, the proportions of tests that rejected *H*_0 _with normal-mixture modeling are all less than the nominal level of 0.05. Further, in all cases, the outcomes obtained by normal-mixture modeling were comparable between the two (assumed) true underlying distributions. The same did not hold for beta-mixture modeling. When the data were not generated according to the beta-mixture scheme, the goodness-of-fit tests were rejected close to or equal to 100% throughout. That is, the best fits of beta-mixtures still could not provide sufficiently close approximations that could pass the goodness-of-fit tests under simulation scenario #1.

**Table 3:** for fecal (mucosal) data using 5, 8, and 12 bins

		True	
	**Fit**	**Beta**	**Normal**

5	Beta	0.12 (0.08)	0.98 (0.01)
	Normal	0.13 (0.09)	0.36 (0.01)

8	Beta	0.00 (0.01)	1.00 (1.00)
	Normal	0.00 (0.01)	0.04 (0.02)

12	Beta	0.02 (0.01)	1.00 (1.00)
	Normal	0.02 (0.00)	0.03 (0.01)

### ICC Comparisons of Fecal and Mucosal Data

Since our findings from the simulation studies suggested that we use a two-component normal-mixture to fit the probit transformed ICC values, we adopted this strategy and utilized it to compare reproducibility under the fecal and mucosal array platforms. We associate the two components of high (and low) ICC values with reproducible (and irreproducible) genes; see the Discussion subsection for more considerations.

We also let, for the fecal and mucosal data, *π*_*LF *_and *π*_*LM *_be the proportions of the mixture components consisting of irreproducible genes, and *μ*_*UF *_and *μ*_*UM *_be the means of the mixture component with higher ICC values. We reported two main studies that were conducted for the purpose of exploring the extent of the distributional differences between the two platforms. Throughout, we used bootstrap methods described in the Methods subsection. The first bootstrap analysis is designed to find the 95% confidence interval for the difference in the proportion of irreproducible genes contained in each data set, *π*_*LF *_- *π*_*LM*_. In the second analysis, we identify the 95% confidence interval for the average difference in the mixture components with higher ICC values, *μ*_*UF *_- *μ*_*UM*_. The bootstrapped 95% confidence intervals for the two studies were (0.06,0.10) for *π*_*LF *_- *π*_*LM*_, and (0.27,0.40) for *μ*_*UF *_- *μ*_*UM*_. As a result, we concluded that while the fecal array had a higher proportion of irreproducible genes, its average ICC values for the reproducible component of genes was a little higher than that obtained from the mucosal platform.

### Outcomes for Analysis of Matched Subset

We now repeat the numerical investigation above but replace the main dataset by the matched subset in which fecal and mucosal measurements were collected from the same genes. The ICC measurements from the matched subset were fitted with a two-component beta-mixture using an EM algorithm, producing the following density estimation for the fecal and mucosal data,  and , respectively:

and

where the additional upper index "s" stands for "subset." We also obtained the following estimated two-component normal-mixture densities,  and , for the probit transformed fecal and mucosal ICC measurements from the matched subset, respectively:

and

**Figure 4:**
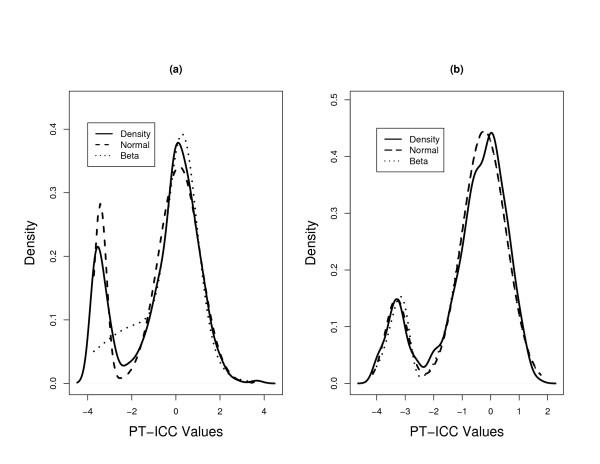
**Density estimates of the probit transformed ICC values for the matched subset for (a) fecal data and (b) mucosal data**. The solid, dashed, and dotted lines correspond to the kernel-based, the normal-mixture based, and the beta-mixture based density estimates.

There were two immediate observations from this sub-study. First, even though the proportions of two components differ from those in the main study, for the PT-ICC values, the estimated parameters correspond fairly well to those from the main study. That is, we obtained almost the same lower and upper components in the normal-mixture modeling as in the main study. On the other hand, the estimated parameter values changed quite dramatically for the beta-mixture modeling. Second, for the mucosal subset, the estimated proportions for the two approaches are almost identical whether the data was fitted by a beta-mixture or a normal-mixture. In fact, by producing a figure equivalent to Figure 3, in Figure 4 we note that the two estimation procedures reach the same conclusion for this estimation (see Figure 4(b)). However, the outcomes produced by beta-mixture modeling remains to be unsatisfactory for the fecal samples. We also obtained the bootstrapped 95% confidence intervals for  and , where the parameters were equivalently defined as in the main study. The two 95% confidence intervals were (0.02, 0.31) and (0.31, 0.63), respectively. They further confirm that, for this matched subset, while the fecal array had a higher proportion of irreproducible genes, its average ICC values for the reproducible component of genes was a little higher than that obtained from mucosal samples.

## Discussion

There are a few points worth making here. The key problem behind the instability of beta-mixture modeling is that one might attempt to estimate the worst component of the mixture distribution with a small proportion of data observed on the boundary. The specifics of simulation scenarios #1 and #2 were based on our analysis of the original subset of ICC values. We expect the same difficulties would be encountered in the beta-mixture modeling if we have a high density of ICC values close to 1 at the upper component. To investigate this conjecture, we conducted an additional simulation study and report the outcomes in the "Additional File 1." We found that the beta-mixture less accurately fit the transformed normal data when the mixture had a high density of values near 1. However, the beta-mixture had no problems fitting transformed normal data resulting from a beta-mixture with no asymptotes at the boundary. There was less distinction between the quality of the fits when the normal-mixture was used to fit PT-ICC data. Again, suggesting that two-component normal-mixture modeling on PT-ICC is a more reliable approach.

Although it is not obvious to interpret the meaning of the estimated parameters, from the normal mixture modeling in Figures 3 and 4, the cut-off between the two mixture components is around -2. This roughly corresponds to the scenario of an ICC = 5%. By pure randomness, even though the true correlation could be zero, one could observe a non-zero sample correlation of 5% or less. From our numerical analyses on the fecal microarray data, the proportion of ICC values less than 5% range from 20% to 28%. The proportion of genes with ICC values less than 5% for the fecal and mucosal samples are 25% and 20%, respectively in the main study, and are 22% and 18%, respectively for the matched study. These numbers again match better with the outcomes from the normal-mixture modeling.

Finally, we conducted another simulation study using the estimated parameters from the matched subset. The exact setup and outcomes are reported in "Additional File 2." For the mucosal subset of ICC values, we find equivalent results between the beta-mixture approach and the normal-mixture approach. However, results from the simulation study show unsatisfactory performances under the scenario of "Data Generated from Normal-mixtures, Fit with Beta-mixtures". Our mucosal matched subset is most likely beta-mixture distributed.

## Conclusion

In this study we have demonstrated that when analyzing ICC values of gene expression levels, it is a better strategy to first probit-transform the ICC values onto the (-8, 8) domain and then to model the PT-ICC values with a normal-mixture model. Through this practice, we were able to obtain outcomes that were less sensitive toward distributional assumptions. We avoided the problem of estimating parameters for a beta distribution which increases to infinity at the boundary. Our investigations suggested that even though there tended to be a higher proportion of genes that had low reproducibility in the fecal array data than in the mucosal array data, the average ICC values for those genes which possessed relatively high ICC values in the fecal data was even a bit higher than the corresponding average observed in the mucosal platform. We also note that the probit transformation strategy enables us to easily adopt the mixture of normal modeling approach that can be carried out by MCLUST packages in R or Splus.

## Methods

### Obtaining ICC Values for Genes on a Microarray Chip

We define a data observation, , to be the gene expression level for gene *g*, subject *i*, treatment *j*, and array *k*. The model for  is given by(1)

for *i *= 1, 2, ..., *I*, *j *= 1, 2, ..., *J*, and *k *= 1, 2, ..., *K*_*ij*_. This describes a microarray experiment where we consider I subjects, J treatments, and *K*_*ij *_arrays for subject *i *under treatment *j*. Also, *μ_j _*is the overall mean for the *j*^*th *^treatment,  is the random effect due to the different subjects, and  is the i.i.d random error. The ICC value for gene *g*, ICC_*g*_, is characterized as(2)

where *G *is the number of genes.

### The Probit Transformation

The probit function[14] is the inverse cumulative distribution function (CDF) of the standard normal distribution. The CDF of the standard normal distribution is often denoted by Φ(*z*), where *z *∈ (-∞, ∞) and the range is (0,1). Specifically,(3)

For *X *in the range of (0,1), the probit transformed values, *Y*, of *X*, are defined as Y = Φ^-1^(*X*), thereby converting (0,1) values to the real line.

### Two-component mixture models

The numerical investigations of ICC and PT-ICC values clearly show that the data comes from a mixture of two populations. When data is modeled by a mixture of two distributions we postulate it as though an observation comes from distribution 1 with probability *p *and from distribution 2 with probability 1 - *π *.

We define *Z*_*i*_, a random indicator variable of the *i*th observation, as

Let *W*_*i *_denote the *i*th observation from the mixture distribution, and assume that

where *f*_1 _and *f*_2 _are the probability density functions of distributions 1 and 2, respectively. The joint distribution of (W,Z) is *f*(*w, z*) = *f*(*w*|*z*)*f*(*z*) and the marginal distribution of W is(4)

That is, for observations {*W*_*i*_|*i *= 1, ..., *n*}, the likelihood function is(5)

### Parameter estimation using expectation-maximization (EM) algorithm

The expectation-maximization (EM) algorithm[15] is an iterative approach for estimation of incomplete data problems. Given starting values of the model parameters, the EM algorithm iteratively updates the estimates until a specified convergence is reached.

#### Mixture of Betas

Ji *et al*. [11] advocate modeling correlation coefficients with beta-mixtures and outline the subsequent EM algorithm. Suppose *y*_1_, . . . *y*_*n *_are *n *independent observations from *f*_*Y *_(*y*|*θ*_*B*_), where *f*_*Y *_is the density of a beta distribution and *θ*_*B *_= (*π, α*_1_, *α*_2_, *β*_1_, *β*_2_). Let the random vector *X *= (*Z, Y*) = {z_*i*_, *y*_*i*_}, where *z_i _*is a 0-1 indicator variable that tells which distribution, the first or the second, the *i*th observation comes from.

In the algorithm, we iteratively perform the "E" and "M" steps with the 'complete' data likelihood function, *L*(*θ*_*B*_|*y*_*i*_), for *θ*_*B *_being(6)

and the corresponding log-likelihood being(7)

In the E-step, **z **is updated with its conditional expectation given the observed data **y**. Consequently,

where the super-index, *k*, denotes the estimates at the *k*th iteration.

In the M-step of the EM algorithm, we use  to estimate the mixing proportion, where

and obtain the maximum likelihood estimates of , , , and  accordingly. The E- and M-steps are iterated until the convergence criteria is met.

The starting values for *α*_1_, *α*_2_, *β*_1_, and *β*_2 _were set to 0.01 and {*z_i_*} was initialized by setting one half of the indicator variables equal to 0 and the other half equal to 1 so that  = 0.50. We utilized the 'optim' function in R to obtain parameter estimates for the two beta density functions. The procedure was repeated until we observed a negligible change in the value of the log-likelihood given in (7).

#### Mixture of Normals

Let *x*_1_, ..., *x*_*n *_be *n *iid observations from *f*_*X*_(*x*|*θ*_*N*_), where *f*_*X *_is the density of a normal distribution and . In order to estimate the parameters for a two-component normal mixture, we use the MCLUST software package for R[16]. MCLUST implements the EM algorithm, equivalent to what what was described for the mixture of betas to carry out the computations of a maximum likelihood approach for normal-mixture models. For model selection, MCLUST determines the number of clusters and the clustering model by maximizing the Bayesian Information Criterion (BIC)[17]. See[16,18] for more details regarding the MCLUST software package.

### Distribution of transformed random variables

#### Generate from Beta, Fit with Normal

Let Y be a random observation from a two-component beta-mixture model with the density function *f*_*B *_given by(8)

where 0 <*π *< 1 and

is the beta density function with shape parameters *α*_*i*_, *β*_*i*_, for *i *= 1, 2. Transform the observations using the probit transformation by letting X = *g*(*Y*) and *g*(·) = Φ^-1^(·). Then the range of *X *becomes (-∞, ∞) and its density function is given by the expression(9)

#### Generate from Normal, Fit with Beta

Let X be a random variable from a two-component normal-mixture model with the density function *f*_*N *_given by(10)

where 0 <*π *< 1 and *φ *(*x*; *μ*_*i*_, ) is the density function of normal random variable with mean *μ*_*i *_and variance , *i *= 1, 2. We define the inverse probit transformation as *Y *= Φ (*X*). The density function of *Y *is given by

### Chi-square goodness of fit

Let *X*_1_, ..., *X*_*n *_be an observed dataset. We divide the range of the data into *k *bins. By comparing the number of observations that fall into a given bin with the expected number of observations for that bin, we are able to use the Pearson's chi-square, *χ*^2^, goodness-of-fit test to assess how well the proposed distribution fits the observed data. The *χ*^2 ^statistic for testing the null hypothesis *H*_0_: The data follow the specified distribution, is(11)

where *O*_*i *_and *E*_*i *_are the observed and expected, respectively frequencies for bin *i*.

To ensure that the expected frequency count is never zero at the tails, we let the first and the last bins to be {*x*|*x *<*X*_(0.025)_} and {*x*|*x *= *X*_(0.975)_}, respectively where *X*_(0.025) _and *X*_(0.975) _are the 2.5th and 97.5th percentiles of the data rounded up and down to the nearest whole numbers. The equal distance bins correspond to the disjoint intervals in between.

If a dataset is fit with a mixture of normal distributions, then the density function defined in (10) is used to determine the expected frequencies. Likewise, we use (9) to calculate expected frequencies when a dataset is fit with a mixture of betas.

### Bootstrap Analysis

We apply bootstrap techniques[19] in order to construct confidence intervals for assessing distributional differences between the fecal and mucosal array platforms. Let *π_LF _*and *π_LM _*be the proportion of irreproducible genes for the fecal and mucosal datasets. The procedure to construct a bootstrap confidence interval for *π*_*LF *_- *π*_*LM *_is as follows:

1. Generate bootstrap samples of size *n*_1 _and *n*_2 _by sampling with replacement from the original *n*_1 _observations of fecal and *n*_2 _observations of mucosal ICC values.

2. Use MCLUST to estimate the parameters of a two-component normal-mixture fitted to each bootstrap sample.

3. Compute .

4. Repeat steps 1 through 3 for I = 299 times, computing .

Once the  are obtained, a (1 - *α*)% bootstrap confidence interval is defined by , where (*α*/2) and (1 - *α*/2) are the *α*/2 and (1 - *α*/2) percentiles of . If we let *μ*_*UF *_and *μ*_*UM *_be the means of the reproducible genes for the fecal and mucosal datasets, then the process for constructing a bootstrap confidence interval for *μ*_*UF *_- *μ*_*UM *_mimics the above procedure, replacing step 3 with "Compute .

## Authors' contributions

NG and NW performed the statistical investigations and prepared the first draft of the manuscript. JRL and NDT are PI and Co-PI, respectively on the grant which resulted in this data set. They were responsible for the design, implementation, and interpretation of the data. RSC's laboratory was responsible for generating the microarrays and LAD was responsible for optimizing the protocol for fecal microarray analysis. All authors read and approved the final version of the manuscript.

## Supplementary Material

Additional file 1 **Simulation scenarios #3 and #4**. These two simulation studie s were designed to show that difficulties would be encountered in a beta-mixture modeling if we have a high density of ICC values close to 1 at the upper component. Scenario #3 represents such a situation while scenario #4 represents a situation where no asymptote is present. Click here for file

Additional file 2 **Simulation scenario mimicking the matched subset data**. These simulation studies were designed to evaluate the study of the matched subsets in which fecal and mucosal measurements were collected from the same genes. Throughout, we let the proportions for the "reproducible" mixture component of the fecal and mucosal datasets to be 0.8 and 0.9, respectively. Otherwise, the mixture parameters reflect those obtained from fitted estimates of the matched subset data. Click here for file
