# Supplementary material for: Evaluation of fecal mRNA reproducibility via a marginal transformed mixture modeling approach
Source: BMC Bioinformatics. 2010 Jan 7;11:13. doi: 10.1186/1471-2105-11-13 (PMC2827371; doi:10.1186/1471-2105-11-13)
Supplement: Additional file 1 — Simulation scenarios #3 and #4. These two simulation studie s were designed to show that difficulties would be encountered in a beta-mixture modeling if we have a high density of ICC values close to 1 at the upper component. Scenario #3 represents such a situation while scenario #4 represents a situation where no asymptote is present. [file 1471-2105-11-13-S1.PDF]

Additional File #1

In what follows, we describe additional simulation studies provided as supplemental information in "*Evaluation of fecal mRNA reproducibility via a marginal transformed mixture modeling approach.*"

Simulation Scenario #3: Beta density values increasing to ∞ at 1
(1) "Generate from beta-mixture, Fit with normal-mixture"

0.6Beta(5.0,0.5)+0.4Beta(1.5,1.5)

(2) "Generate from normal-mixture, Fit with beta-mixture"

0.6N(1.8,1.0)+0.4N(0.3,0.9)

Simulation Scenario #4: No Asymptote
(1) "Generate from beta-mixture, Fit with normal-mixture"

0.6Beta(1.5,4.0)+0.4Beta(6.0,2.0)

(2) "Generate from normal-mixture, Fit with beta-mixture"

0.6N(-0.7,0.4)+0.4N(0.7,0.3)

In order to get the above mixture distributions, we first devised beta-mixtures to model the given scenarios. A density plot showing the behavior of these two beta-mixtures is provided in Figure A1.1. In order to obtain the fitted normal-mixture, we generated one ICC dataset under each scenario, transformed the data via probit-transformation, and fit the PT-ICC to a normal-mixture model.

For the beta-mixture which increases to ∞ at 1, we carried out the simulation study as follows:

Data Generated from Beta-mixtures, Fit with Normal-mixtures
(i) Generate $Y_1, \ldots, Y_n$ from $\tilde{f}_B^i = 0.6\ Beta(5.0, 0.5) + 0.4\ Beta(1.5, 1.5)$.
(ii) Transform $Y_1, \ldots, Y_n$ using the probit transformation and fit the PT-ICC measurements with a two-component normal-mixture model.

Data Generated from Normal-mixtures, Fit with Beta-mixtures
(i) Generate $X_1, \ldots, X_n$ from $\tilde{f}_N^i = 0.6\ N(1.8, 1.0) + 0.4\ N(0.3, 0.9)$.
(ii) Transform $X_1, \ldots, X_n$ using the inverse probit transformation and fit the transformed data with a two-component beta-mixture model.

We repeated each simulation s=250 times for sample size n=1000 and used the EM algorithm to obtain the estimates of corresponding parameters. The

steps above were repeated for the beta-mixture with no asymptotes at the boundaries by generating beta random variables from $\tilde{f}_B^n = 0.6\, Beta(5.0, 0.5) + 0.4\, Beta(1.5, 1.5)$ and the normal random variables from $\tilde{f}_N^n = 0.6\, N(-0.7, 0.4) + 0.4\, N(0.7, 0.3)$.

A1.1 Analysis

When comparing the true parameters with the estimates obtained from the fit of the assumed distribution, we find greater deviation from the truth when fitting transformed normal random variables with a beta-mixture that increases to infinity at 1. For both datasets, we find there to be less bias when normal-mixture modeling is used to fit probit-transformed ICC data. Although there is less discrepancy between the simulation schemes for the beta-mixture with no asymptote at the boundaries, it is still better to fit the normal-mixture modeling. When the beta-mixture increases to infinity at 1, the average estimated upper proportion is 0.54 when a normal-mixture is used to fit the data, compared to 0.48 when a beta-mixture is used to the fit the data. The truth is 0.60.

Table A1.1: Summary statistics of simulation scenarios #3 and #4 when data are generated from beta-mixtures and fit with normal-mixtures. Monte Carlo mean, bias, standard deviation, and square-root MSE (RMSE) of upper mixture proportion μ_U, upper mixture mean μ_U and variance σ_U^2, and lower mixture mean μ_L and variance σ_L^2 are reported.

		Generate from Beta, Fit with Normal				
Dataset		$\hat{\pi}_U$	$\hat{\mu}_U$	$\hat{\sigma}_U^2$	$\hat{\mu}_L$	$\hat{\sigma}_L^2$
**Scenario #3**	Truth	0.600	1.763	0.777	-0.007	0.623
	Mean	0.542	1.674	1.021	0.300	0.857
	Bias	-0.058	-0.089	0.244	0.307	0.234
	Std Dev	0.063	0.217	0.150	0.273	0.148
	RMSE	0.086	0.235	0.286	0.411	0.277
**Scenario #4**	Truth	0.600	-0.722	0.375	0.756	0.248
	Mean	0.574	-0.692	0.379	0.660	0.298
	Bias	-0.026	0.030	0.004	-0.096	0.050
	Std Dev	0.052	0.227	0.050	0.270	0.050
	RMSE	0.058	0.229	0.050	0.287	0.071

Table A1.2: Summary statistics of simulation scenarios #3 and #4 when data are generated from normal-mixtures and fit with beta-mixtures. Monte Carlo mean, bias, standard deviation, and square-root MSE (RMSE) of upper mixture proportion μ_U, upper mixture mean μ_U and variance σ_U^2, and lower mixture mean μ_L and variance σ_L^2 are reported.

		Generate from Normal, Fit with Beta				
Dataset		$\hat{\pi}_U$	$\hat{\mu}_U$	$\hat{\sigma}_U^2$	$\hat{\mu}_L$	$\hat{\sigma}_L^2$
**Scenario #3**	Truth	0.600	1.763	0.777	-0.007	0.623
	Mean	0.455	1.800	0.967	0.695	1.297
	Bias	-0.145	0.037	0.190	0.702	0.674
	Std Dev	0.066	0.293	0.258	0.245	0.280
	RMSE	0.159	0.295	0.320	0.743	0.730
**Scenario #4**	Truth	0.600	-0.722	0.375	0.756	0.248
	Mean	0.540	-0.811	0.324	0.618	0.322
	Bias	-0.060	-0.089	-0.051	-0.138	0.074
	Std Dev	0.058	0.074	0.042	0.087	0.050
	RMSE	0.083	0.116	0.066	0.163	0.089

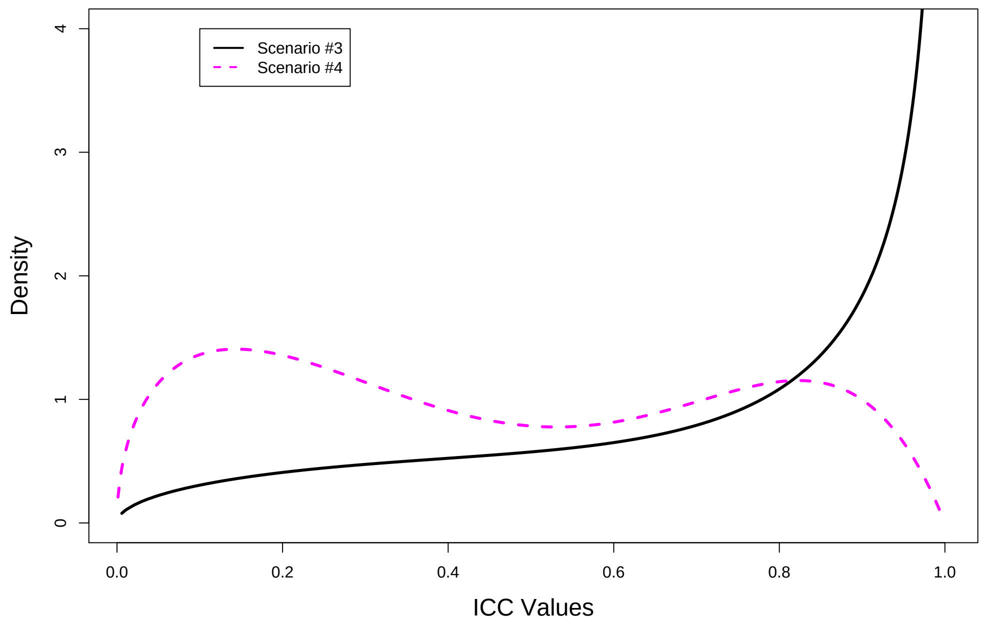

Figure A1.1: *The density of the fitted two-component beta-mixtures for scenarios #3 and #4 which model a beta-mixture increasing to infinity at 1 (solid line) and a beta-mixture with no asymptotes at the boundaries (dashed line).*
