# Supplementary material for: Evaluation of fecal mRNA reproducibility via a marginal transformed mixture modeling approach
Source: BMC Bioinformatics. 2010 Jan 7;11:13. doi: 10.1186/1471-2105-11-13 (PMC2827371; doi:10.1186/1471-2105-11-13)
Supplement: Additional file 2 — Simulation scenario mimicking the matched subset data. These simulation studies were designed to evaluate the study of the matched subsets in which fecal and mucosal measurements were collected from the same genes. Throughout, we let the proportions for the "reproducible" mixture component of the fecal and mucosal datasets to be 0.8 and 0.9, respectively. Otherwise, the mixture parameters reflect those obtained from fitted estimates of the matched subset data. [file 1471-2105-11-13-S2.PDF]

Additional File #2

In what follows, we describe the simulation studies mimicking the matched subsets as provided in "*Evaluation of fecal mRNA reproducibility via a marginal transformed mixture modeling approach.*" Throughout, we let the proportions for the reproducible-gene component of the fecal dataset and mucosa dataset to be 0.8 and 0.9, respectively, while we let the parameter values to be given by the estimates obtained from the matched subset data.

Simulation Scenario #5: Fecal Data
(1) "Generate from beta-mixture, Fit with normal-mixture"

0.8Beta(2.7,1.7)+0.2Beta(0.2,0.6)

(2) "Generate from normal-mixture, Fit with beta-mixture"

0.8N(0.2,0.9)+0.2N(-3.5,0.1)

Simulation Scenario #6: Mucosa Data
(1) "Generate from beta-mixture, Fit with normal-mixture"

0.9Beta(1.3,1.8)+0.1Beta(0.8,910.2)

(2) "Generate from normal-mixture, Fit with beta-mixture"

0.9N(-0.2,0.6)+0.1N(-3.3,0.2)

In order to get the above mixture distributions, we fit the original ICC values to a beta distribution and fit PT-ICC values to a normal-mixture. A density plot showing the behavior of these two beta-mixtures is provided in Figure A2.1.

For the beta-mixture of the fecal data, we carried out the simulation study as follows:

Data Generated from Beta-mixtures, Fit with Normal-mixtures
(i) Generate $Y_1, \ldots, Y_n$ from $\tilde{f}_B^f = 0.8Beta(2.7, 1.7) + 0.2Beta(0.2, 0.6)$.
(ii) Transform $Y_1, \ldots, Y_n$ using the probit transformation and fit the PT-ICC measurements with a two-component normal-mixture model.

Data Generated from Normal-mixtures, Fit with Beta-mixtures
(i) Generate $X_1, \ldots, X_n$ from $\tilde{f}_N^f = 0.8N(0.2, 0.9) + 0.2N(-3.5, 0.1)$.
(ii) Transform $X_1, \ldots, X_n$ using the inverse probit transformation and fit the transformed data with a two-component beta-mixture model.

We repeated each simulation s=250 times for sample size n=1000 and used the EM algorithm to obtain the estimates of corresponding parameters. The steps above were repeated for the beta-mixture of the mucosa data by generating beta random variables from $\tilde{f}_B^m = 0.9Beta(1.3, 1.8)+0.1Beta(0.8, 910.2)$ and the normal random variables from $\tilde{f}_N^m = 0.9N(-0.2, 0.6)+0.1N(-3.3, 0.2)$.

A2.1 Analysis

Table A2.1: Summary statistics of simulation scenarios #5 and #6 when data are generated from beta-mixtures and fit with normal-mixtures. Monte Carlo mean, bias, standard deviation, and square-root MSE (RMSE) of upper mixture proportion μ_U, upper mixture mean μ_U and variance σ_U^2, and lower mixture mean μ_L and variance σ_L^2 are reported.

		Generate from Beta, Fit with Normal				
Dataset		$\hat{\pi}_U$	$\hat{\mu}_U$	$\hat{\sigma}_U^2$	$\hat{\mu}_L$	$\hat{\sigma}_L^2$
**Scenario #5**	Truth	0.800	0.347	0.442	-1.546	3.732
	Mean	0.813	0.341	0.425	-1.685	3.739
	Bias	0.013	-0.006	-0.017	-0.139	0.007
	Std Dev	0.024	0.028	0.033	0.277	0.509
	RMSE	0.027	0.028	0.037	0.310	0.509
**Scenario #6**	Truth	0.900	-0.252	0.606	-3.325	0.164
	Mean	0.893	-0.250	0.59	-3.258	0.235
	Bias	-0.007	0.002	-0.016	0.067	0.071
	Std Dev	0.023	0.027	0.037	0.228	0.314
	RMSE	0.024	0.027	0.040	0.238	0.322

Table A2.2: Summary statistics of simulation scenarios #5 and #6 when data are generated from normal-mixtures and fit with beta-mixtures. Monte Carlo mean, bias, standard deviation, and square-root MSE (RMSE) of upper mixture proportion μ_U, upper mixture mean μ_U and variance σ_U^2, and lower mixture mean μ_L and variance σ_L^2 are reported.

		Generate from Normal, Fit with Beta				
Dataset		$\hat{\pi}_U$	$\hat{\mu}_U$	$\hat{\sigma}_U^2$	$\hat{\mu}_L$	$\hat{\sigma}_L^2$
**Scenario #5**	Truth	0.800	0.347	0.442	-1.546	3.732
	Mean	0.632	0.322	0.693	-2.194	2.690
	Bias	-0.168	-0.025	0.251	-0.648	-1.042
	Std Dev	0.111	0.094	0.145	0.850	1.672
	RMSE	0.201	0.098	0.290	1.068	1.970
**Scenario #6**	Truth	0.900	-0.252	0.606	-3.325	0.164
	Mean	0.760	-0.128	0.492	-1.884	2.092
	Bias	-0.140	0.124	-0.114	1.441	1.928
	Std Dev	0.071	0.049	0.063	0.694	0.905
	RMSE	0.157	0.134	0.130	1.600	2.130

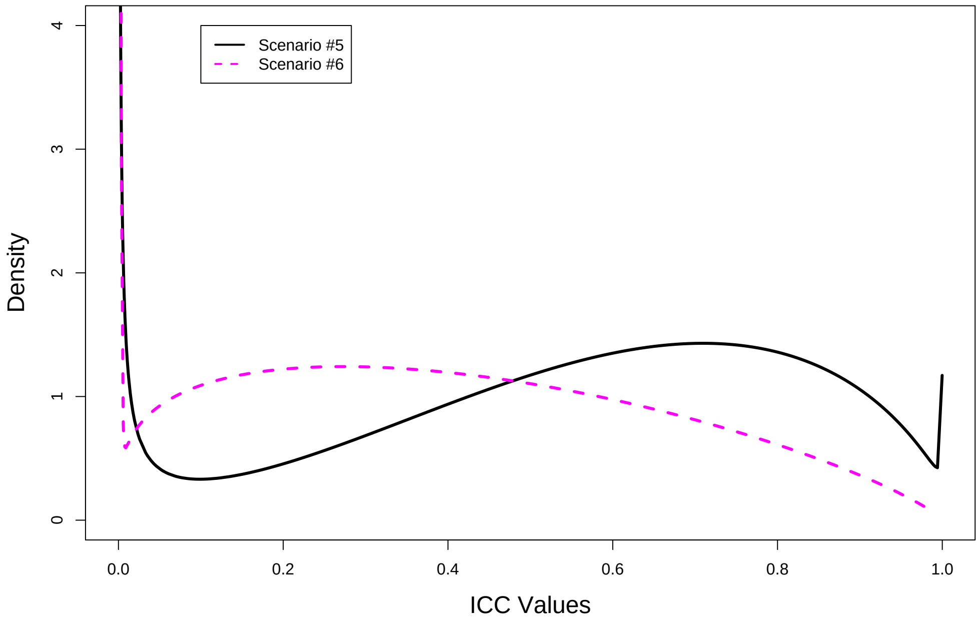

Figure A2.1: *The density of the fitted two-component beta-mixtures for scenarios #5 and #6 which model the estimated beta-mixture of the fecal (solid line) and mucosa (dashed line) ICC values.*
